# Atherogenic subfractions of lipoproteins in the treatment of metabolic syndrome by physical activity and diet – the RESOLVE trial

**DOI:** 10.1186/1476-511X-13-112

**Published:** 2014-07-11

**Authors:** Frédéric Dutheil, Guillaume Walther, Robert Chapier, George Mnatzaganian, Bruno Lesourd, Geraldine Naughton, Julien Verney, Anne Fogli, Vincent Sapin, Martine Duclos, Agnès Vinet, Philippe Obert, Daniel Courteix, Gérard Lac

**Affiliations:** 1 Laboratory of Metabolic Adaptations to Exercise in Physiological and Pathological conditions (AME2P, EA3533), Blaise Pascal University, Clermont-Ferrand, France; 2 School of Exercise Science, Australian Catholic University, East Melbourne, VIC, Australia; 3 Sport Medicine and Functional Exploration, University Hospital CHU G. Montpied, Clermont-Ferrand, France; 4 Occupational Medicine, University Hospital CHU G. Montpied, Clermont-Ferrand, France; 5 Laboratory of Pharm-Ecology Cardiovascular (EA4278), Avignon, France; 6 Faculty of Health Sciences, Australian Catholic University, East Melbourne, VIC, Australia; 7 Geriatrics, Faculty of Medicine, Clermont-Ferrand, France; 8 Biochemistry, University Hospital CHU G. Montpied, Clermont-Ferrand, France

**Keywords:** Metabolic syndrome, Obesity, Cardiovascular risk, Framingham, Physical activity, Diet, Nutrition, Apolipoproteins

## Abstract

**Background:**

We aimed to comprehensively evaluate lipoprotein profile including lipid particle size following a lifestyle intervention in metabolic syndrome (MetS) volunteers and to assess the associations between lipoprotein subfractions and carotid-intima-media-thickness (CIMT) – a surrogate indicator of atherogenesis.

**Methods:**

100 participants (50–70 years) from the RESOLVE trial, underwent a one-year follow-up beginning with a three-week residential program combining high exercise volume (15-20 h/week), restrictive diet (-500 kcal/day), and education. For baseline references, 40 aged-matched healthy controls were recruited. Independent associations between subfractions of lipoproteins and CIMT were evaluated using a generalized estimating equations model accounting for variation in correlations between repeated measures. The lipoprotein subfractions profile was assessed using Lipoprint® electrophoresis allowing to separate: the very low-density lipoprotein (VLDL) fraction, then the intermediate-density lipoprotein (IDL) C, B and A, the low-density lipoprotein (LDL) with subfractions 1 and 2 as large LDL and subfractions 3 to 7 as small dense LDL (sdLDL), and the high density lipoprotein (HDL) subfractions categorized into large, intermediate, and small HDL. Apolipoproteins A1 and B were also measured.

**Results:**

78 participants completed the program. At baseline, apolipoproteins B/A1, VLDL, sdLDL and small HDL were higher in MetS than in healthy controls; IDL, LDL size, large and intermediate HDL were lower. Despite time-related regains during the follow-up, lipoprotein subfractions traditionally involved in cardiovascular risk, such as sdLDL, improved immediately after the residential program with values closest to those of healthy controls. CIMT improved throughout the lifestyle intervention. Using a generalized estimating equations model, none of the subfractions of lipoproteins nor apolipoproteins were linked to CIMT.

**Conclusions:**

Lipoprotein subfractions traditionally involved in CVR, decreased after the 3-week residential program. During a 12 month follow-up, the time-related regains remained closer to the values of healthy controls than they were at baseline. CIMT improved throughout the lifestyle intervention. However, we failed to demonstrate a link between some lipoprotein subfractions and the atherogenicity directly measured from the wall thickness of arteries (CIMT). Further investigations are required to explore the atherogenicity of lipoprotein subfractions.

**Trial registration:**

NCT00917917

## Introduction

Cardiovascular disease is the main cause of morbidity and mortality in individuals with metabolic syndrome (MetS) [1]. Cardiovascular disease risk factors (CVR) include an excess of body fat, promoting dyslipidemia, with reduced high-density lipoprotein cholesterol (HDL) and increased low-density lipoprotein cholesterol (LDL) [2]. A low level of HDL is regarded as a sensitive discriminator of atherogenicity and is one among the five criteria selected by the International Diabetes Federation (IDF) to characterize MetS [3]. More specifically, lipoproteins can be differentiated into subfractions using electrophoresis. LDL was categorized into subfractions 1 to 7, relative to decreasing size and increasing density. Among them, the small dense subfractions 3 to 7 (sdLDL), not routinely assessed in clinical practice, are presumed to be more atherogenic than larger LDL particles [4,5]. The notion of HDL functionality was more recently introduced, with an increased CVR associated with a decreased HDL size [6-8]. Relationships also emerged between atherogenesis and other lipoproteins classes, very low-density lipoprotein (VLDL) [9] and intermediate-density lipoprotein (IDL) [10]. Moreover, apolipoproteins (Apo) are the structural protein constituting the lipoproteins [11]. The atherogenicity of MetS is possibly mediated by elevated ApoB [12], with the proatherogenic/antiatherogenic ratio ApoB/ApoA1 being strongly linked to CVR [11]. The relationship between lipoprotein profiles and atherogenesis has a strong clinical focus. Early detection of CVR may prevent a later diagnosis of cardiovascular disease by using strategies such as lifestyle interventions [13,14]. The Framingham score [15], although commonly used to estimate the CVR, may not permit the early risk detection available from profiling the lipoprotein subfractions.

However, 1) previous studies that assessed the relationship between CVR and some subfractions of lipoproteins were mainly cross-sectional [16-20], 2) the role of each subfractions is debated [21]. Even for the more robust theory on atherogenicity of sdLDL, a predominance of very large rather than sdLDL has been reported with increased CVR [22], 3) The few studies reporting longitudinal changes of lipoprotein subfractions following a lifestyle intervention (diet, physical activity) describe relatively acute [23] or short term responses [13,24-26], 4) lack direct measures of atherogenicity inside the walls or arteries [13,23-26], 5) provide limited selections of lipoprotein subfractions [13,23-26], and 6) are not reported in populations with MetS [13,23-26].

Therefore, we aimed to describe long-term changes in the profile of the lipoprotein subfractions among metabolic syndrome volunteers from the RESOLVE trial [27] who participated in a lifestyle intervention (diet and physical activity). A secondary aim was to assess the associations between lipoprotein subfractions and carotid-intima-media-thickness (CIMT) – a surrogate indicator of atherogenesis.

## Results

### Participants

One hundred participants (43 males, 57 females, mean age 59.4 ± 5.0 years, 91.4 ± 12.9 kg, body mass index (BMI) 33.4 ± 4.1 kg/m^2^) with MetS were recruited into this one-year study commencing with a three-week residential program. At baseline, blood levels were 5.5 ± 1.5 mmol/l for glucose, 6.3 ± 0.8% for HbA1c, and 140.3 ± 14.2 mmHg for systolic and 84.1 ± 9.7 mmHg for diastolic blood pressure. Seventy-eight completed the whole intervention [27]. Participants who dropped out of the program and those who completed had similar baseline characteristics with similar cardiovascular risk profiles. The mean compliance scores regarding diet and exercise during the at-home follow-up were 61.7 ± 24.3% between the 21^st^ day (D20) and the 3^rd^ month (M3), 52.8 ± 24.31% between M3 and the 6^th^ month (M6), and 49.1 ± 23.5% between M6 and the 12^th^ month (M12). The intervention had positive effects on weight (-3.5 ± 0.2 kg at D20, -6.8 ± 0.4 kg at M3, -6.7 ± 0.7 kg at M6, and -6.0 ± 0.8 kg at M12, from baseline) and on weight-related changes [27]. The matched healthy control group was composed of 26 males and 24 females with mean age of 58.0 ± 4.7 years and BMI of 22.4 ± 6.5 kg/m^2^.

### Lipoprotein subfractions

#### Baseline

**Figure 1:**
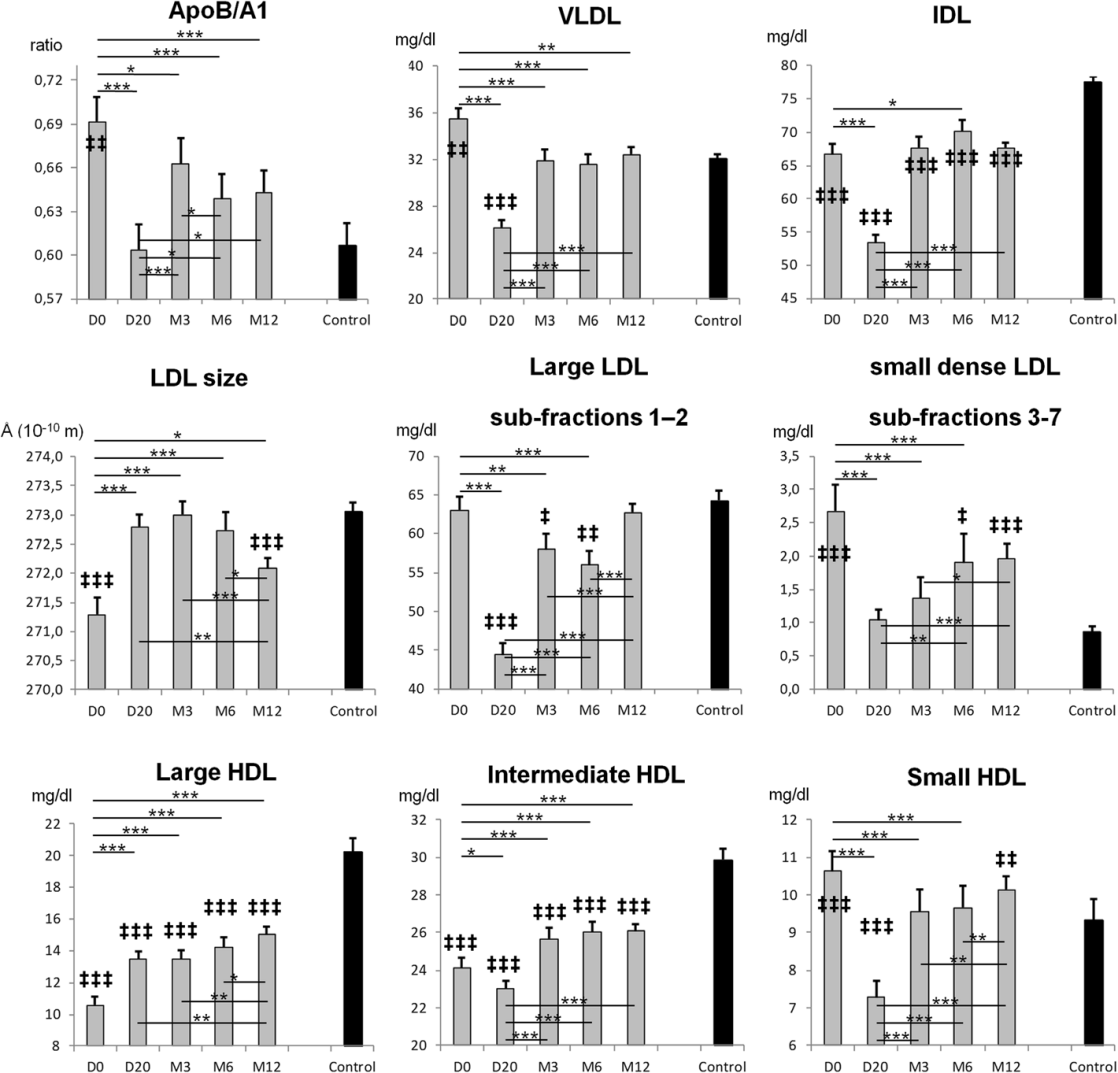
**Apolipoprotein B/A1 ratio and other lipoproteins sub-fractions profile using quantimetrix Lipoprint™ system.** The very low-density lipoprotein (VLDL) fraction, then the intermediate-density lipoprotein (IDL), the low-density lipoprotein (LDL) size which can distinguish the large LDL sub-fractions 1–2, non atherogenic, and the small dense LDL (sdLDL) sub-fractions 3–7, atherogenic, and the high-density lipoprotein (HDL) sub-fractions (large, intermediate and small). *: p < .05; **: p < .01; ***: p < .001 between different time of measurements for MetS participants. ‡: p < .05; ‡‡: p < .01; ‡‡‡: p < .001 between MetS participants and controls.

At baseline, individuals with MetS differed significantly from their matched healthy controls in all subfractions of lipoproteins and apolipoproteins with the exception of large LDL. Specifically, ApoB/A1, VLDL, sdLDL and small HDL were higher in MetS than in healthy controls; IDL, LDL size, large and intermediate HDL were lower in MetS than in healthy controls (Figure 1).

#### Follow-up

The greatest time-related changes were observed at the end of the residential program (D20) and a progressive return to baseline values occurred during the at-home follow-up for ApoB/A1, VLDL, IDL, Large and sdLDL, and small HDL. Large HDL increased throughout the study. Even if only ApoB/A1 and VLDL failed differ from healthy values of controls at M12, LDL size, and large and intermediate HDL were closer to values of healthy controls at M12 than at baseline (Figure 1).

### Secondary outcomes

**Figure 2:**
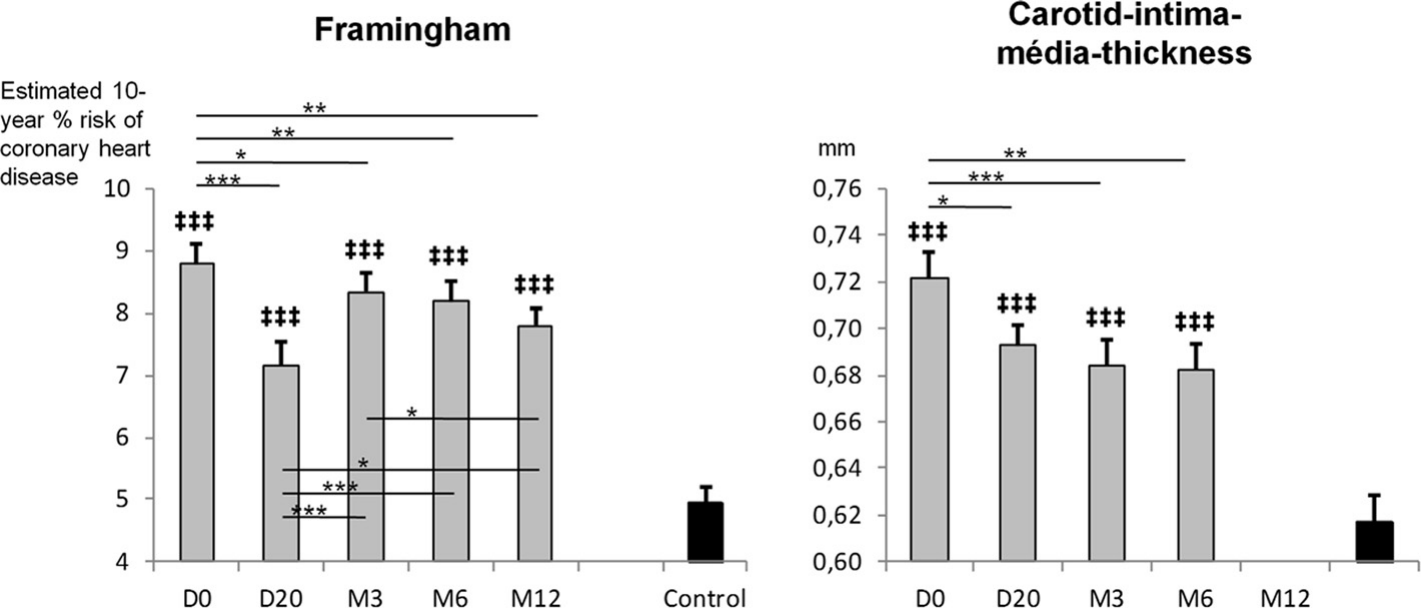
**Framingham score and carotid-intima-media thickness in patients with MetS undertaking a lifestyle intervention.** *: p < .05; **: p < .01; ***: p < .001 between different time of measurements for MetS participants. ‡‡‡: p < .001 between MetS participants and controls.

CIMT and the Framingham score decreased, but remained higher than baseline values of healthy controls (Figure 2). Other general outcomes (clinical and biological parameters) have been previously described and followed the same pattern [27].

### Correlations

CIMT was correlated with large HDL (r = -.142, p = .004) and IDL (r = -.183, p < .001). The Framingham score was correlated with large (r = -.253, p < .001) and intermediate HDL (r = -.226, p < .001), VLDL (r = -.178, p < .001), IDL (r = .315, p < .001), large (r = .360, p < .001) and sdLDL (r = .127, p = .012).

The change in CIMT between M6 and D0 correlated with changes in ApoB/A1 between M6 and D0 (r = -.265, p = .018). Changes in the Framingham score between M6 and D0 correlated with the changes in Large HDL (r = -.343, p < .001) and intermediate HDL (r = -.222, p = .031) between M6 and D0.

### Multivariable analyses

Change over time in CIMT and Framingham score and the impact of covariates on these changes were separately modeled using a multivariable generalized estimating equations model that also accounted for variation in the correlation between the repeated measurements (Table 1). The multivariable model that controlled for the variables listed in Table 1 showed that only type 2 diabetes was independently associated with an increase in CIMT levels. Similarly, there was a significant visit effect when controlling for all variables listed in Table 1. The CIMT level decreased for each visit (time of measurement = effect of physical activity and diet). None of the subfractions of lipoproteins nor apolipoproteins were linked to CIMT. On the other hand, Framingham score increased with age, type 2 diabetes, and ApoB and it decreased with ApoA1, large HDL and Intermediate HDL.

**Table 1:** Association of CIMT or Framingham score with lipoprotein subfractions: generalized estimating equations multivariable analyses

***Models with ApoA1 and ApoB***				
	**Outcome: CIMT**		**Outcome: Framingham score**	
**Variables**	**Coefficient (95% CI)**	**P-value**	**Coefficient (95% CI)**	**P-value**
Age, continuous	0.00 (-0.00 – 0.00)	0.173	**0.11 (0.07 – 0.16)**	**<0.001**
Body Mass Index, continuous	0.00 (-0.00 – 0.00)	0.441	0.05 (-0.00 – 0.11)	0.057
Diabetes Mellitus, yes	**0.05 (0.02 – 0.08)**	**0.001**	**3.03 (2.52 – 3.54)**	**<0.001**
ApoA1, continuous	-0.00 (-0.05 – 0.04)	0.959	**-3.25 (-4.13 – -2.37)**	**<0.001**
ApoB, continuous	0.01 (-0.04 – 0.05)	0.754	**6.139 (5.32 – 6.96)**	**<0.001**
Visit	**-0.01 (-0.02 – -0.00)**	**0.019**	-0.07 (-0.20 – 0.06)	0.288
***Models with individual lipoprotein subfractions (without ApoA1 or ApoB)***				
Age, continuous	0.00 (-0.00 – 0.00)	0.170	**0.10 (0.06 – 0.15)**	**<0.001**
Body Mass Index, continuous	0.00 (0.01 – 0.00)	0.569	0.01 (-0.04 – 0.06)	0.718
Diabetes Mellitus, yes	**0.05 (0.02 – 0.08)**	**0.002**	**2.94 (2.45 – 3.43)**	**<0.001**
VLDL	-0.00 (-0.00 – 0.00)	0.942	0.01 (-0.02 – 0.32)	0.675
IDL-C	-0.00 (-0.00 – 0.00)	0.163	0.03 (-0.01 – 0.07)	0.189
IDL-B	0.00 (-0.00 – 0.00)	0.193	0.02 (-0.03 – 0.07)	0.426
IDL-A	-0.00 (-0.00 – 0.00)	0.827	**0.05 (0.02 – 0.08)**	**0.002**
Large LDL (1-2)	0.00 (-0.00 – 0.00)	0.442	**0.036 (0.02 – 0.05)**	**<0.001**
Small dense LDL (3-7)	0.00 (-0.00 – 0.00)	0.962	-0.01 (-0.06 – 0.04)	0.764
Large HDL	0.00 (-0.00 – 0.00)	0.631	**-0.10 (-0.16 – -0.05)**	**<0.001**
Intermediate HDL	-0.00 (-0.01 – 0.00)	0.187	**-0.13 (-0.19 – -0.07)**	**<0.001**
Small HDL	0.00 (-0.00 – 0.01)	0.497	-0.03 (-0.10 – 0.05)	0.484
Visit	**-0.01 (-0.02 – -0.00)**	**0.015**	-0.08 (-0.21 – 0.05)	0.245

All models also controlled for sex, compliance, and exercise intensity type.

Significant data (p<.05) are highlighted in bold.

## Discussion

### Principal findings

The major findings showed that lipoprotein subfractions traditionally involved in CVR, such as sdLDL, were markedly improved after the 3-week residential program. Then, the time-related regains remained closer to the values of healthy controls. CIMT improved throughout the lifestyle intervention. However, we failed to demonstrate a link between some lipoprotein subfractions and the atherogenicity directly measured from the wall thickness of arteries (CIMT).

### Improvement and time related regain of lipoprotein subfractions

Even with normal levels of total LDL, cross-sectional studies historically showed a link between sdLDL and CVR [16-20]. This link persisted independently of other lipid parameters [4,28,29]. In our study, sdLDL dropped at D20 reaching control values, and despite a subsequent regain, remained lower at M12 than at baseline. The LDL size followed a similar pattern, which could possibly be related to the high volume and intensity of exercise during the residential program [30,31]. Even if there is some evidence to support the hypothesis that certain forms of large LDL particles may be atherogenic [22], our participants with MetS had the same values of large LDL as the healthy controls at baseline and at the end of the study. It can be noted that sdLDL and large LDL followed the same change. HDL is commonly considered as a protective agent against cardiovascular disease, which explains why low levels of HDL are included among the five components defining MetS [3]. However, the screening of the HDL subclasses has added interest to the notional importance of HDL functionality. The favorable role of HDL must be limited to the large fractions only, the small fractions having conversely a pejorative role [6-8]. Our intervention also supported a favorable result for HDL subfractions. Small HDL decreased, with again a marked transient drop at D20, and large subclasses rose, strongly between D0 and D20, and marginally from D20 to M12. Within other lipoprotein subfractions, similar observations are made for intermediate subclasses and VLDL in response to physical activity and diet [13,32]. The improvement in VLDL levels, classically elevated in MetS [33], seems to be notable because of its contribution to endothelium damages [34]. The proatherogenic/antiatherogenic ratio ApoB/ApoA1 shows stronger links to MetS and may be a better risk discriminator than the single proatherogenic measurement (ApoB) [11]. Between baseline and D20, MetS individuals reached the ApoB/A1 values of the healthy controls. In this study we also describe for the first time the long-term one-year changes in levels of the whole lipoprotein subfractions in patients with MetS [13,23-26].

### Lipoprotein subfractions and atherogenicity

To contribute to the existing debate on subfractions and atherogenicity [21], currently mainly based on cross-sectional studies [16-20], we added a direct measure of atherogenicity inside the walls or arteries [13,23-26] in our long-term follow-up design. The CIMT is a direct measure of atherogenesis [35], whereas the Framingham score is a probability of a cardiovascular event established from a cluster of indexes: age, smoking status, type 2 diabetes, blood pressure, LDL and HDL-cholesterol [15]. Despite observed improvements in the Framingham score and carotid-intima-media-thickness, values failed to reach the control group, but remained better than baseline. The Framingham score decreased significantly from baseline to M12, with a marked and transient drop at D20. Overall, the improvement was gradual from baseline to M6 for CIMT (data not collected at M12) with the difference acutely significant by D20. Therefore, it is possible that the time-delayed changes in CIMT were due to the anatomical nature of this marker which is less dynamic and more structural (arterial wall) than other blood related changes. Conversely, the Framingham score reacted quickly to changes in lifestyle and were mainly linked to LDL and HDL cholesterol, based on the method of its calculation.

In order to minimize the probability of false positives which increased with the number of correlations, we ran a multivariable generalized estimating equations model that controlled for various confounders and also accounted for variation in correlation between the repeated measures. To verify our findings, we also modeled the Framingham score. As expected, we found that ApoA1 and ApoB were significantly associated with the Framingham score. ApoB is the structural protein for the atherogenic lipoproteins (VLDL, IDL and LDL) responsible for transporting lipid from the liver and gut to peripheral tissues, and ApoA1 is the major structural protein for HDL, responsible for excess cholesterol in peripheral tissues carried back to the liver for excretion [11]. Thus, results are tightly coherent since they are based on the formula to calculate the Framingham score. The strong links with type two diabetes demonstrated similar plausibility. Associations between CIMT, and type two diabetes are well established [36]. However, we failed to demonstrate a link between the lipoprotein subfractions and CIMT. Further investigations are required to explore the atherogenicity of lipoprotein subfractions.

### Strengths and limitations

Our study presents some major strengths: run-in design; community-based long term intervention; direct measure of atherogenicity inside the walls or arteries; description of changes in all lipoprotein subfractions; the use of an appropriate model to assess the independent associations of each of the lipoprotein subfractions with CVR; MetS participants; a large sample size; the originality is also arisen because such training volumes (15–20 h/week) have seldom been investigated in obesity over 12 months.

However, this study has some limitations. CIMT was not measured at M12 for financial reasons. Other mechanistic cardiovascular measures assessing CVR are not yet available. A group without physical activity could have provided opportunities to distinguish the effects of physical activity from the diet. Implementing our intervention in health practice is costly and our high volume training protocol (15 hours per week) may prove difficult to comply with in usual practice.

## Conclusion

In conclusion, lipoprotein subfractions traditionally involved in CVR, decreased after the 3-week residential program. During a 12 month follow-up, the time-related regains remained closer to the values of healthy controls than they were at baseline. CIMT improved throughout the lifestyle intervention. However, we failed to demonstrate a link between some lipoprotein subfractions and the atherogenicity directly measured from the wall thickness of arteries (CIMT). Further investigations are required to explore the atherogenicity of lipoprotein subfractions.

## Methods

### Participants

The general methodology of RESOLVE has been described previously [27]. Briefly, participants were eligible if they were aged between 50 and 70 years, suffering from MetS [3], living a sedentary lifestyle, stable body weight and unchanged medical treatment over the previous 6 months, no hepatic, renal or psychiatric diseases, nor cardiovascular or endocrine diseases except those defining MetS, no use of medications known to alter body weight, no restricted diet in the previous year, and able to complete a maximal exercise tolerance test. For baseline references, aged-matched healthy controls were recruited with no disease/medication and no parameter of the MetS. All participants provided written informed consent. The study received the approval from the ethics committees of St Etienne’s University Hospital, France.

### Baseline assessments

**Figure 3:**
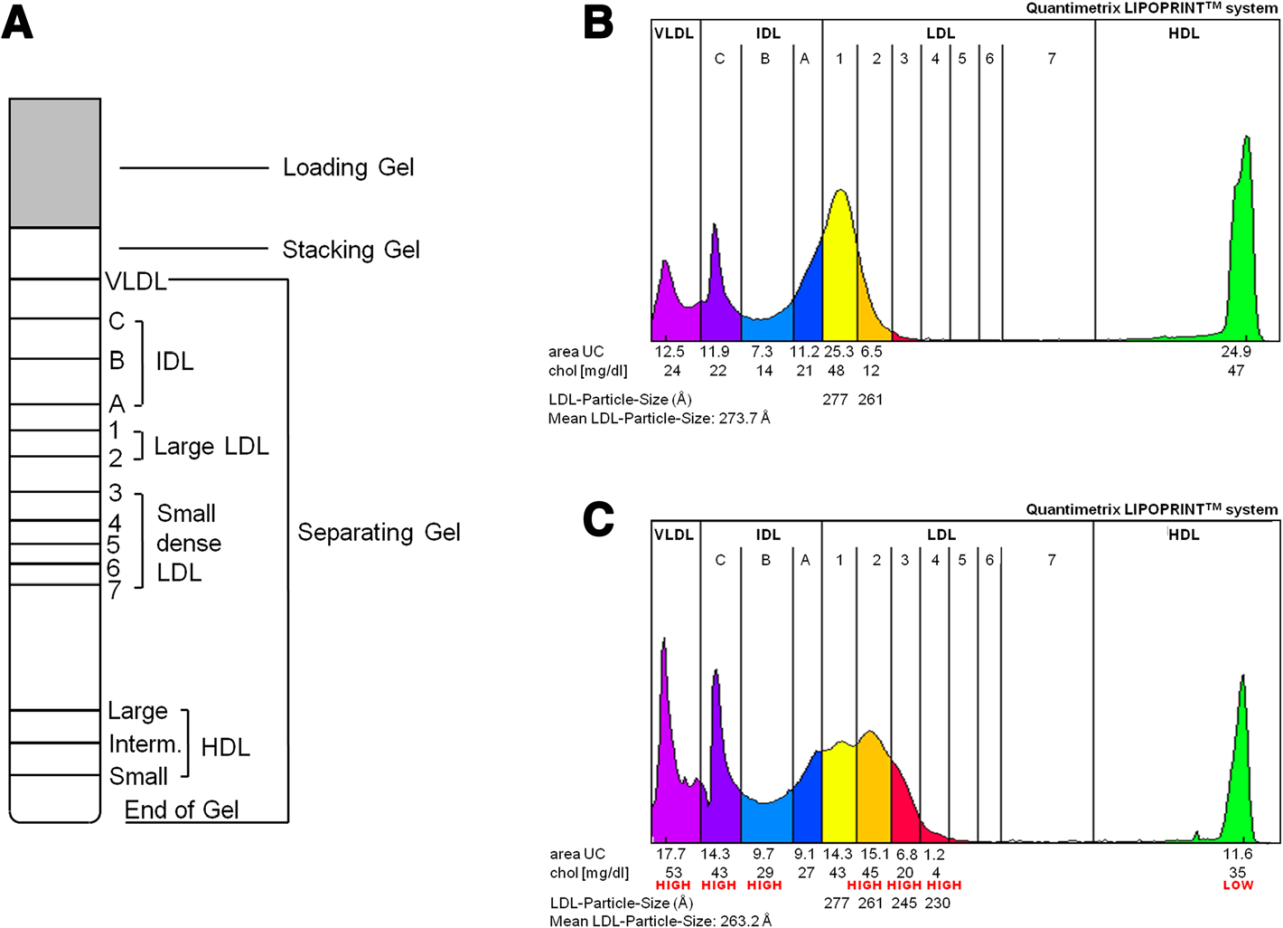
Range of lipoproteins sub-fractions on the separating gel (A), with a normal profile (B) and a pathological profile (C), using quantimetrix Lipoprint™ system.

The lipoprotein subfractions profile was assessed using Lipoprint® electrophoresis (Quantimetrix Inc., Redondo Beach, California). This method is based on electrophoresis of lipid stained serum (Sudan black) in non-denaturing gel gradient of polyacrylamide [37]. Different subfractions were identified by their migration distance (Figure 3). Each lipoprotein subfraction has a specific electrophoretic mobility with the VLDL fraction at the origin of the separating gel, then the IDL, the LDL, and the HDL fraction at the end of the gel. The Lipoprint system can identify 7 subfractions of LDL. The diameter of the LDL particles at the cut-off point separating subfractions 1–2 (large and commonly less atherogenic) from subfractions 3–7 (small dense and atherogenic) was 251 Å [37]. Similarly, HDL subfractions can be categorized into large, intermediate, and small subfractions [38]. The system includes tube gels and proprietary data analysis software to determine the subfraction concentrations based on area under the curve [38]. Coefficient of variation (CV) varies according to the level of lipoproteins. Intra- and interassay ranges of CV were respectively 5.58 – 7.28% and 7.12 – 9.40% for VLDL, 2.94 – 11.14% and 4.73 – 13.63% for IDL, 1.05 – 1.52% and 1.26 – 1.57% for LDL, and 1.87 – 2.84% and 2.49 – 4.69% for total HDL. For more information, please see the technical informations available on the Lipoprint® Quantimetrix® website.

Fasting blood samples were drawn between 7.00 and 7.30 a.m., aliquoted and stored at -80°C until analyses. Basic biological assays were performed in the biochemistry laboratory of the University Hospital (Clermont-Ferrand, France), including ApoA1 and ApoB.

Individual risk factor scores were summed to determine the 10-year absolute risk of cardiovascular disease using Framingham score [15].

The common carotid artery structure was evaluated using a high-resolution B-mode ultrasound (MyLab30, Esaote SpA, Firenze, Italy) The CIMT was defined as the distance from the leading edge of the lumen-intima interface to the leading edge of the media-adventitia interface of the far wall. The CIMT of the left CIMTs was measured automatically by dedicated software (MyLab desk 9.0, Esaote, Florence, Italy) according to the Mannheim consensus [35].

### Follow-up assessments

All baseline assessments were repeated at 21 days (D20), 3 months (M3), 6 months (M6) and 12 months (M12), with the exception of the CIMT which was not measured at M12.

### First stage of intervention: a 3-week residential program

Throughout the residential program, MetS participants underwent a daily diet restriction (-500 kcal/day) with protein accounting for 15 to 20% of the total energy intake (1.2 g/kg/day), lipids 30 to 35%, and carbohydrates the remainder. Participants had to exercise using endurance training (90 min daily: aquagym, cycling or walking) and resistance training (90 min × four days a week). Resistance training consisted of 8 exercises with free weights and traditional muscle building equipment. Participants were coached individually, and heart rate was monitored by Polar™ S810. Participants also attended lectures dealing with MetS, nutrition, cooking and exercise to support the sustainability of their new lifestyle on returning home.

### Second stage of intervention: a 1-year at-home follow-up

At the end of first stage of intervention (D20), MetS participants were asked to maintain the lifestyle (diet and physical activity) experienced during the residential program, with follow-up at M3, M6 and M12. Dietary and exercise practices during this period were assessed with the use of a compliance score determined on the basis of the number of food questionnaires returned and the number of training sessions undertaken per week. The overall compliance score was the mean of these two scores (nutrition and physical activity) [27].

### Statistical analysis

Data are presented as mean ± SD. Statistical analyses were performed using SPSS (v19, SPSS Inc., Chicago, IL, USA) and Stata software (v12, Stata-Corp, Texas, USA). Normality of distribution was assessed by the Shapiro-Wilk test. The primary focus of the analyses was the 12-month change in lipoprotein subfractions’ serum levels. Changes over time were tested by a one-way ANOVA with repeated-measures, with the use of Bonferroni post-hoc test. Correlation matrices between lipoprotein subfractions and CVR were constructed using the non parametric Spearman test. In order to minimize the probability of false positives when numerous correlations are calculated, the independent associations of each of the lipoprotein subfractions with CVR (Framingham score or CIMT) were further assessed using a multivariable generalized estimating equations model that controlled for age, gender, body mass index, presence of diabetes, participant’s compliance and exercise intensity while accounting for multiple correlations between the repeated measures. The descriptive characteristics of those who dropped out of the program were also summarized. We also performed a descriptive statistical analysis for participants who dropped out to compare their characteristics with those who completed the program. Significance was set at the p < .05 level.

## Abbreviations

Apo: Apolipoproteins; CIMT: Carotid-intima-media-thickness; CV: Coefficient of variation; CVR: Cardiovascular disease risk factors; D0: Day 0 corresponding to baseline; D20: The 21^st^ day at the end of the residential program; HDL: High-density lipoprotein cholesterol; IDF: International Diabetes Federation; IDL: Intermediate-density lipoprotein; LDL: Low-density lipoprotein-cholesterol; M3: The 3^rd^ month; M6: The 6^th^ month; M12: The 12^th^ month; MetS: Metabolic syndrome; sdLDL: Small dense subfractions 3 to 7; VLDL: Very low-density lipoprotein.

## Competing interests

The authors have no competing interests.

## Authors’ contributions

FD has participated as a PhD student and main investigator. FD, BL, DC, PO, GW, AV and GL contributed to the conception of the protocol. FD recruited all patients. MD and FD did maximal exercise testing. FD and GL did aliquoting of blood samples. PO, GW and AV performed cardiovascular measurements. VS and AF measured all biologic data. RC was responsible for the residential program. BL supervised daily diet and managed physical activity. FD and GM conducted the statistical analysis. FD and GL drafted the manuscript. GN and GM revised the manuscript. All authors read and approved the final manuscript. FD is the guarantor of this study and takes full responsibility for the work as a whole.
